# Assessing the potential of sputtered gold nanolayers in mass spectrometry imaging for metabolomics applications

**DOI:** 10.1371/journal.pone.0208908

**Published:** 2018-12-12

**Authors:** Pere Ràfols, Dídac Vilalta, Sònia Torres, Raul Calavia, Bram Heijs, Liam A. McDonnell, Jesús Brezmes, Esteban del Castillo, Oscar Yanes, Noelia Ramírez, Xavier Correig

**Affiliations:** 1 Department of Electronic Engineering, Universitat Rovira i Virgili, Tarragona, Spain; 2 Spanish Biomedical Research Centre in Diabetes and Associated Metabolic Disorders (CIBERDEM), Madrid, Spain; 3 Center for Proteomics & Metabolomics, Leiden University Medical Center, Leiden, The Netherlands; 4 Department of Pathology, Leiden University Medical Center, Leiden The Netherlands; 5 Fondazione Pisana per la Scienza ONLUS, Pisa, Italy; 6 Institut d’Investigació Sanitària Pere Virgili, Tarragona, Spain; University of Campinas, BRAZIL

## Abstract

Mass spectrometry imaging (MSI) is a molecular imaging technique that maps the distribution of molecules in biological tissues with high spatial resolution. The most widely used MSI modality is matrix-assisted laser desorption/ionization (MALDI), mainly due to the large variety of analyte classes amenable for MALDI analysis. However, the organic matrices used in classical MALDI may impact the quality of the molecular images due to limited lateral resolution and strong background noise in the low mass range, hindering its use in metabolomics. Here we present a matrix-free laser desorption/ionization (LDI) technique based on the deposition of gold nanolayers on tissue sections by means of sputter-coating. This gold coating method is quick, fully automated, reproducible, and allows growing highly controlled gold nanolayers, necessary for high quality and high resolution MS image acquisition. The performance of the developed method has been tested through the acquisition of MS images of brain tissues. The obtained spectra showed a high number of MS peaks in the low mass region (*m/*z below 1000 Da) with few background peaks, demonstrating the ability of the sputtered gold nanolayers of promoting the desorption/ionization of a wide range of metabolites. These results, together with the reliable MS spectrum calibration using gold peaks, make the developed method a valuable alternative for MSI applications.

## Introduction

Classic histopathological analysis, in which the visual inspection of stained tissue sections is used for the identification of specific morphological regions and minute structures in the tissue, is one of the essential tools in medical diagnosis. Although often successful, in ambiguous cases the pathologist is not always capable of determining the correct diagnosis using histopathological methods alone. Complementary techniques that elucidate the chemical composition of those tissues allow a more accurate diagnosis and thus aid the pathologist in these cases. In recent years, mass spectrometry imaging (MSI) has emerged as a useful tool for the untargeted, and spatially correlated molecular analysis of clinical tissues, providing chemical information directly from the tissue [1,2].

The most widely used ionization technique in MSI is matrix-assisted laser desorption/ionization (MALDI) [3], where an organic matrix is applied on a sample surface to promote the desorption/ionization of the analytes (e.g. proteins, lipids and metabolites). Nevertheless, ionization techniques such as desorption electro-spray ionization (DESI) are becoming more widespread since its introduction in MSI [4]. DESI is carried out by spraying solvent charged droplets directly onto the tissue section. The impact of the charged droplets with the sample is capable to start the desorption process of the analytes [5]. In contraposition to other ionization techniques, DESI operates at ambient pressure and no organic matrix or ionization material is needed. This simplifies the sample preparation and allows the use of DESI in vivo. Nevertheless, DESI cannot achieve the high lateral resolutions which MALDI is capable of, because the focalization of the laser cannot be matched with a solvent sprayer [5].

Classical MALDI-matrix application techniques may introduce artefacts like compound diffusion, deteriorating the lateral resolution of the image [6], and/or inhomogeneities during deposition over the tissue and/or co-crystallization leading to increased differences in pixel-to-pixel ion intensities [7]. It is also known that some highly volatile organic matrices like 2,6-dihydroxyacetophenone (DHA) and dithranol evaporate during their time in the high vacuum ion source of the mass spectrometer, resulting in measurement artefacts over long data acquisition times [8]. To overcome some of these problems, matrix sublimation has been introduced for matrix deposition allowing higher spatial resolution analyses, as it is a solvent-free matrix deposition method and therefore results in smaller sized matrix crystals and less lateral diffusion of analytes [9]. The sublimation approach solves the compound delocalization problem, however MALDI organic matrices still introduce many low-weight MS signals which interfere severely with the MS peaks of endogenous low weight compounds, complicating the application of MSI to metabolomics studies [7].

Matrix-free LDI-MS have been proposed as an attractive alternatives to analyze low molecular weight metabolites. Commonly used matrix-free techniques are surface-assisted laser desorption/ionization (SALDI), in which ionization is supported by the surface of the target plate [7,10–15], and nanostructure-initiator mass spectrometry (NIMS) [16], which uses molecules of an initiator compound trapped in nanostructured surfaces promoting the ionization of the metabolites. Moreover, metal (Au, Ag, Pt, etc.) and metal oxide (WO_3_, TiO_2_, Fe_3_O_4_, ZnO, etc.) nanoparticles and nanolayers, frequently called nanoparticle-assisted LDI (nano-PALDI) have also been used for the LDI-MS analysis of biomolecules [7]. In this context, gold nanoparticles are likely the ideal substrate because they present high stability, can be easily functionalized [17,18], are able to absorb the UV light emitted by the laser and effectively transfer this absorbed energy to the metabolites promoting its absorption and providing a source of ionization. Several studies have used gold nanoparticles for the analysis of biofluids by LDI-MS, and for MSI applications achieving an effective ionization of low mass range metabolites with very low background signal [19–25]. In these studies, gold nanoparticles were deposited on the tissues by mixing them with organic matrices or solvents. This “wet” deposition of gold does not prevent the potential lateral diffusion of the metabolites and the inhomogeneous distribution of the gold nanoparticles. As an alternative to this, AuNPET has been introduced as a simple and effective Au enhanced MALDI target plate which enables the direct MSI acquisition of imprinted samples [26]. This method has been proven very effective and straightforward to obtain metabolomics information from fingerprints [26] and more recently to study metabolite spatial distributions in garlic [27]. However, the AuNPET workflow is vulnerable to smudging of the spatial detail during the imprinting step, and so is less suitable for softer samples such as tissues, or for higher spatial resolution investigations. In order to achieve a very high lateral resolution, it has also been proposed to directly analyze a tissue section mounted on a ITO-coated glass slide without using any material to promote the LDI process[28]. However, this methods demands a high laser power and a spectrometer with high sensitivity like an FTICR. To overcome this, sputter deposition, which is a solvent-free and reproducible deposition technique, would allow the deposition of high purity, homogeneous metal or metal oxide nanolayers onto biological tissues while avoiding molecular delocalization associated with solvent-based application methods or tissue imprinting imperfections. In a previous publication by Dufresne *et al*. sputter deposition of silver was used prior to the MSI of olefins from tissue sections [29]. This study demonstrated the viability of sputter depositions for MSI metabolomics applications, with high spatial resolution (down to 5 μm). More recently, a sodium deposition followed by a sputtered gold layer has been introduced as a powerful method for the analysis of triacylglycerols [30]. Furthermore, the characteristic gold and silver peaks and clusters can be used for internal mass calibration along the different *m/z* regions of the obtained spectrum [29–31].

**Fig 1:**
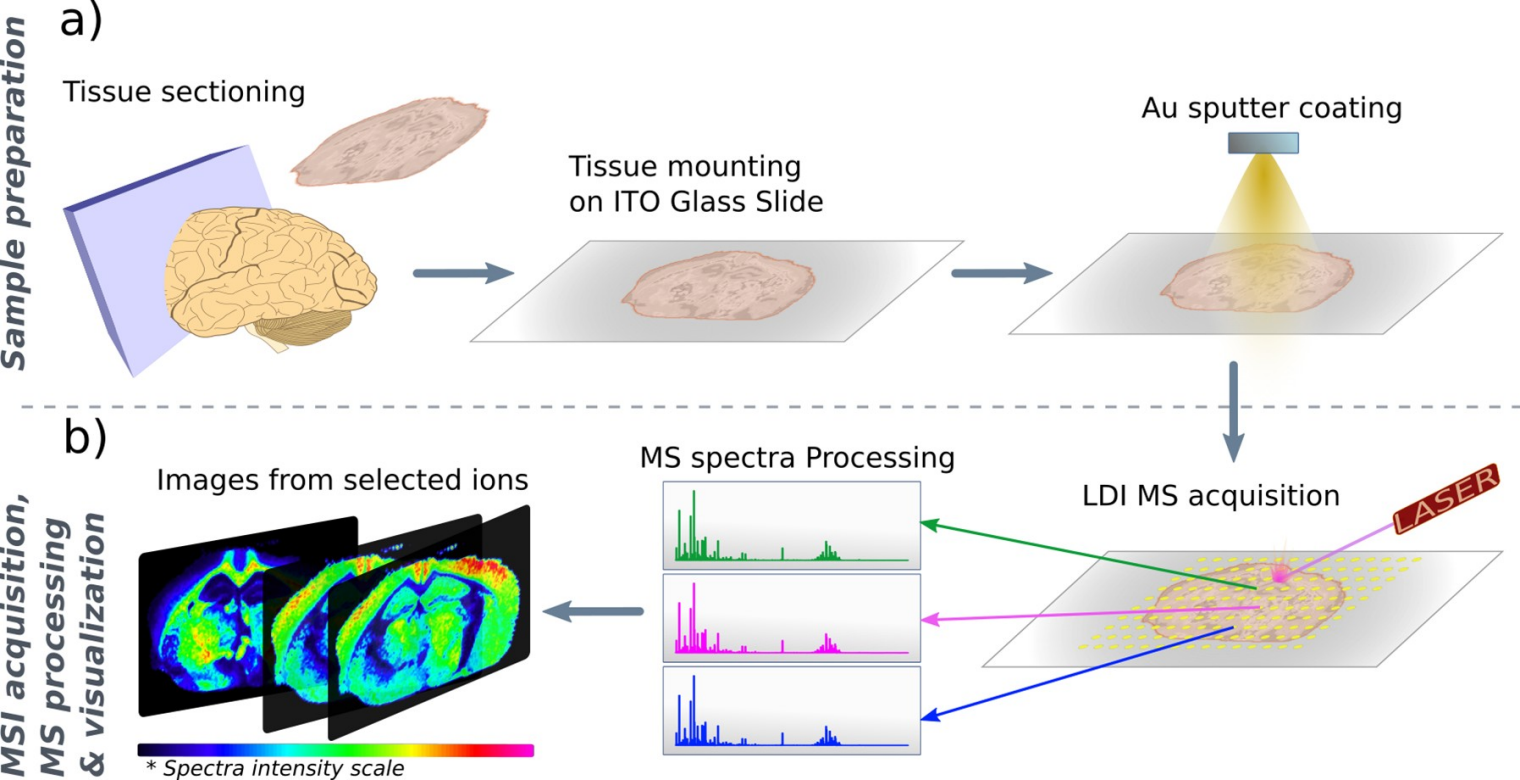
Experimental workflow of the developed gold nanolayer-assisted LDI-MSI method. **(A)** Sample preparation, including sectioning of 10μm-thick sections, tissue mounting on indium-tin oxide-coated (ITO) glass slides and the tissue coating with gold nanoparticles by sputtering. **(B)** Summary of LDI-MSI acquisition, spectral pre-processing, image reconstruction and visualization.

Hence, in this study we present the application of gold nanolayers deposited by sputtering directly onto the tissue section to obtain metabolomic MS images of animal tissues by LDI-MS. In comparison with silver, gold has only one stable isotope, thus reducing the number of peaks and facilitating the detection of trace compounds. Gold ionizes polar and heavier metabolites more effectively [26], and provides highly stable nanolayers. The experimental workflow used in this study is summarized in Fig 1 and includes the cryosectioning of the fresh frozen tissues into thin sections (10 μm), the mounting of these tissue sections on conductive indium-tin oxide-coated (ITO) glass slides and the coating of the sections with gold nanoparticles using sputter deposition. In this study we demonstrated the performance of our LDI methodology using fresh frozen tissues. However, the same gold coating technique can be applied to formalin-fixed and paraffin-embedded (FFPE) tissues since the MALDI organic matrix deposition stage can be directly replaced by the sputter gold coating procedure. In case of preparing FFPE tissues for sputter gold coating it is highly advised to follow specific paraffin removal protocols in order to preserve the MS signals of low-weight molecules[32]. After the spatially correlated LDI-MS acquisition of a spectrum at each pixel, spectra were processed and the images of the molecular distributions reconstructed and visualized. In this study, we report the optimization of the sputter deposition conditions, based on the ionization efficiency of the gold nanolayers by using mice liver sections and the optical and morphological characterization of the deposited gold nanolayers. The viability of the optimized gold nanolayers was checked by the acquisition of metabolomics MSI data from mouse brain tissue, and comparison to negative controls to show the increase in MS performance obtained by the presented gold coating method. This work contributes to the validation of the novel sputtered Au approach as a reliable methodology for low *m/z* range MSI applications. Nevertheless, this preliminary study should be further developed in the future by applying it to specific MSI applications.

## Materials and methods

### Materials

Indium tin oxide (ITO)-coated glass slides were obtained from Bruker Daltonics (Bremen, Germany). The gold-target (purity grade > 99.995%) used for sputtering was obtained from Kurt J. Lesker Company (Hastings, England). The reagents and solvents for staining were hematoxylin and HPLC grade xylene supplied by Sigma-Aldrich (Steinheim, Germany) and ethanol (96% purity, supplied by Scharlau, Sentmenat, Spain).

### Sample preparation

The liver tissue used for gold-sputtering optimization was obtained from C57BL/6 mice provided by Dr M. Teresa Colomina, professor of Psychobiology at the Research Center for Behavioral Assessment (CRAMC) of the Universitat Rovira i Virgil. The brain tissue used for the example of MSI metabolites assignation was obtained from C57BL/6 mice of 6 months old. These tissues were provided by Professor Martins-Green’s research group at the Cell Biology Department of the University of California Riverside. The tissues were snap frozen at -80°C after collection and stored and shipped at this temperature until analysis. Animal experimental protocols were approved by the University of California, Riverside, Institutional Animal Care and Use Committee (IACUC).

The brain tissues used for the high-lateral resolution MSI analysis were obtained from three month-old, male, C57BL/6J mice. These tissues were obtained from Leiden University Medical Center where all the high lateral resolution experiment was carried out. The brains were excised, flash-frozen on dry-ice and stored at -80°C until analysis. All experiments were approved by the Animal Experiment Ethics Committee of Leiden University Medical Center.

For MSI acquisition, the tissues were sectioned at -20°C into 10 μm thick sections using a Leica CM-1950 cryostat (Leica Biosystems Nussloch GmbH) located at the Centre for Omics Sciences (COS) of the University Rovira i Virgili and mounted on ITO coated slides by directly placing the glass slide at ambient temperature onto the section. To remove residual humidity, samples where dried in a vacuum desiccator for 15 minutes after tissue mounting.

### Gold sputter coating

Gold nanolayers were deposited onto the 10 μm tissue sections using a sputtering system ATC Orion 8-HV (AJA International, N. Scituate, MA, USA). An argon atmosphere with a pressure of 30 mTorr was used to create the plasma in the gun. The working distance of the plate was set to 35 mm. The deposition times were determined from the deposition rate, which is directly proportional to the layer thickness. Since deposition times used in this study were very short, the substrate temperature remained cold during the deposition, thereby avoiding degradation of the tissue metabolites. The final optimized sputtering conditions for MSI were at ambient temperature, using RF mode at 60 W for 35 s. For negative control MSI experiments tissues were not subjected to gold sputter coating (0 s deposition time).

### Sample characterization

Reflectance measurements of the gold-coated tissues were carried out with a Lambda-950 spectrophotometer, equipped with deuterium and tungsten lamps (Perkin-Elmer, Waltham, MA, USA) scanning in the 250 to 800 nm wavelength range.

Morphology of the gold layer was characterized by transmission electronic microscopy (TEM) using a JEOL 1011 microscope (Jeol, Peabody, MA, USA). A TEM grid was used to deposit a gold layer using the optimized conditions described above.

### LDI-MS acquisition

MSI data used for the Au-layer optimization and characterization were acquired using a MALDI TOF/TOF UltrafleXtreme instrument with SmartBeam II Nd:YAG/355 nm laser from Bruker Daltonics, also at the COS facilities. Acquisitions were carried out using the medium and large laser spot size settings, operated at 2 kHz at an attenuated power of 60%, collecting a total of 500 shots per pixel.

High spatial resolution MSI data were recorded using a MALDI TOF/TOF rapifleX with SmartBeam 3D II Nd:YAG/355 nm laser from Bruker Daltonics, located at Leiden University Medical Center (LUMC). The laser was operated at 10 kHz collecting 200 shots per pixel.

Pixel sizes from 10 to 1000 μm were used during the optimization. The TOF mass spectrometer was operated in positive ion, reflectron mode, with a digitization rate of 1.25 GHz, *m/z* range 70 to 1200 Da achieving a calculated resolving power of about 12k at 1000 Da, with a manually optimized extraction delay. The spectrometer was calibrated prior to MSI data acquisition using [Au]^+^ peaks as reference masses. Following the LDI-MSI experiment, the sections were stained with hematoxylin.

### Spectra pre-processing and image visualization

MSI data was acquired using the Flex-software suite (v3.0 Bruker Daltonics). Each MSI dataset was exported to the XMASS data format using instrument manufacturer software packages (FlexImaging and Compass export) and a custom script. The data stored in XMASS was converted to a custom format based on segmented matrices storage highly optimized for processing large MSI datasets in R language [33]. Mass spectra were aligned using a novel unlabeled method developed to handle our custom data format efficiently and which is included in our rMSIproc package (http://github.com/prafols/rMSIproc)[34]. After alignment, the whole dataset shared the same mass axis and, therefore mass calibration was applied to the whole dataset by only calibrating the mean spectrum. Following this method, masses were calibrated using gold peaks as reference: 196.9666, 393.9331, 590.8997, 787.8662 and 984.8328 Da. Moreover, *m/z* 96.9223 and 112.8962 associated with [KNaCl]^+^ and [K_2_Cl]^+^ were also used as mass reference peaks to better calibrate the low-mass range ions [26]. In order to show the actual performance of the gold layer, no normalization was performed. MSI datasets were explored manually to select a set of peaks localized on different morphological structures. This exploration stage was accomplished using our dedicated graphical user interface included in the rMSI R package, specially developed to rapidly explore MSI data [33]. MSI image reconstruction and visualization was also performed with the same in-house software package.

### Metabolite identifiaction

MS peaks were obtained using an in-house peak picking algorithm included in the rMSIproc R package with S/N>5. The obtained list of MS peaks was matched with the HMDB 3.0 [35] database within a tolerance of 20 ppm and the possible ion adducts: H, Na, K and NH_4_. In order to obtain a list of possible metabolites, the obtained search results were filtered using the biological information of molecules provided by the HMDB. We have also used the information provided by the HMDB to highlight the putative identified metabolites that have previously been reported in brain tissues.

## Gold nanolayer optimization and characterization

### Sputter coating optimzation for LDI

The sputtered deposition of gold nanoparticles has been optimized to achieve the highest LDI-MS signal intensities at the lowest laser fluencies. The gold nanolayer deposited on the tissue must provide enough gold nanoparticles to promote the desorption/ionization of the metabolites, but also thin enough to enable the laser to reach the tissue-gold interface. Moreover, the deposited gold nanolayer must ensure the correct identification of the gold MS peaks to enable in-situ mass calibration using these peaks.

To optimize the layer thickness we have used liver sections from C57/BL mice. We have selected a liver tissue from a healthy mouse for the optimization steps because it usually presents high biological homogeneity at the spatial resolution used for MSI analysis, facilitating the comparison of the performance of the different gold layers in a real sample. To further ensure the comparability of the tests, the various gold layers were deposited over consecutive liver sections. Moreover, each acquisition was performed on identical regions of tissue, which were selected by optical inspection of the liver sections. Then, a wide random walk of 1000 μm per pixel was used in order to obtain an averaged spectrum of each laser shot.

As a starting point we tested three different Au nanolayers designed to cover a broad range of Au thicknesses. Once an approximate optimal layer was obtained, the next step was to fine-tune the sputter coating time to fine-tune the Au thickness. Two modes can be selected for gold sputtering: direct current (DC), which is the fastest method, and radio frequency (RF), which provides higher control over the deposited gold layers. Since the desired Au nanolayer must be a thin layer according to previous studies [29,30], we performed all Au depositions operating the sputtering system in RF mode to better control the tissue coating process. The first three deposition times tested as first thickness exploration were 25, 100 and 300 s at 60 W and ambient temperature.

The laser attenuation reported in FlexImaging was adjusted for each one of the three sputtered layers. This laser attenuation setting is part of the user interface, designed to give the average user fine control over the laser powers commonly used in MALDI experiments (0% corresponds to the laser power offset, and 100% to the laser power offset + laser power range, both of which can be found in the instrument specific settings). The laser fluence varies approximately linearly with the attenuation throughout this range. Here, we report the laser attenuation value to compare the laser fluence used for each sputtered layer. This laser attenuation parameter was adjusted in order to achieve the highest MS peak intensities in the *m/z* 700–900 range for each gold layer. Based on their molecular masses, the metabolites that can be found in this mass range are likely to correspond to phosphatidylcholines and triacylglycerides, compounds of high biological relevance, but easily fragmented. Therefore, during the laser fluence adjustment we monitored the intensity of *m/z* 184, which corresponds to the head group fragment of the phosphatidylcholines. We selected the best performing laser power fluence for each Au layer to obtain a good tradeoff between peak intensity and molecular fragmentation. The optimal laser powers were found to be 60, 70 and 75% for the 25, 100 and 300 s sputter coating times respectively. The thicker layers required higher laser power, suggesting that the ionization efficiency is lower for thicker layers and were more prone to suffer fragmentation due to higher laser power.

**Fig 2:**
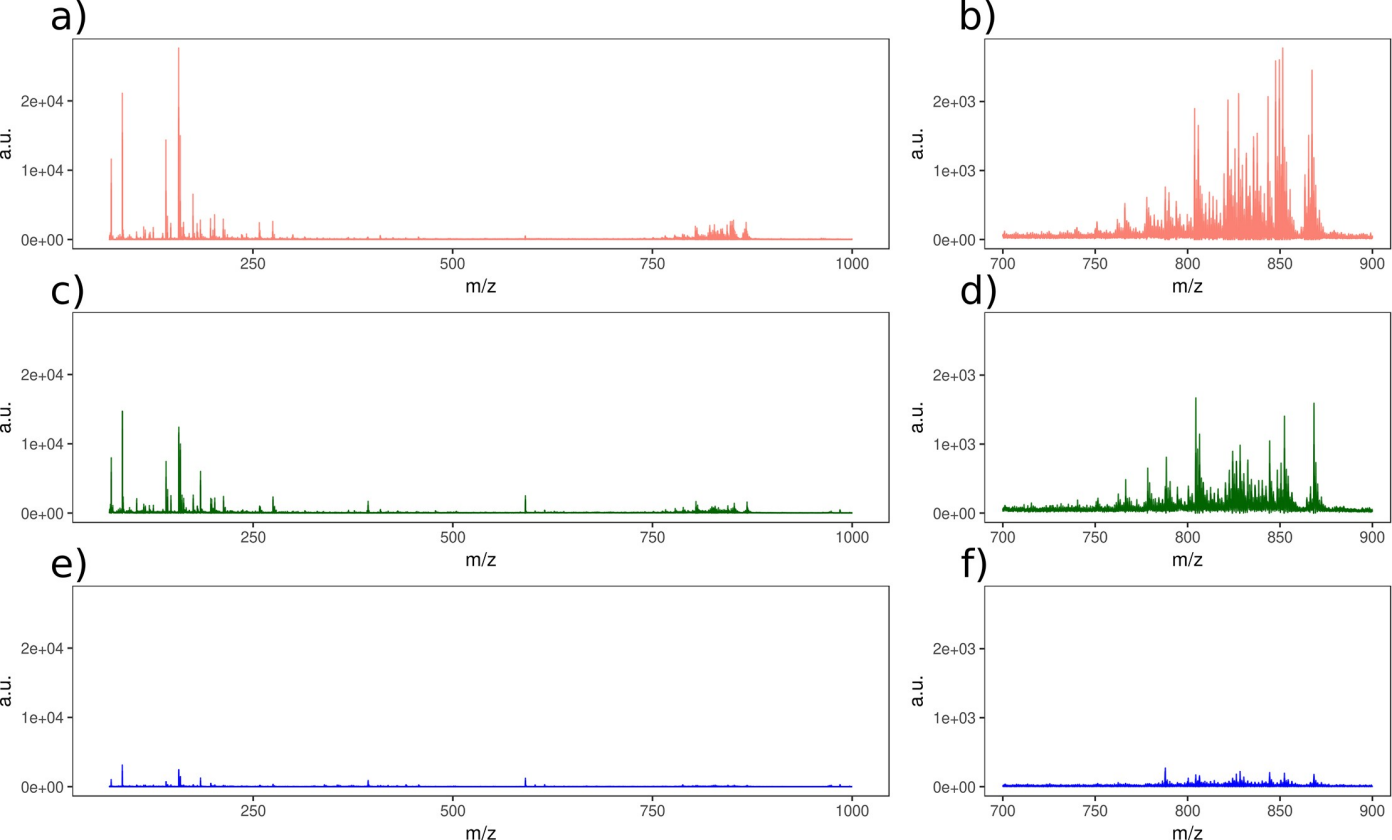
**Average spectra of mouse liver sections obtained with each of the three tested gold layers: (A)** 25 s Au coating time at a laser power of 60%, **(C)** 100 s Au coating time at laser power of 70%, **(E)** 300 s Au coating time at laser power of 75%, **(B)**, **(D)** and **(F)** figures plot the *m/z* spectrum between 700 and 900 Da to illustrate the performance of the tested gold layers in a specific area of the spectrum.

Fig 2 shows the average spectra of the liver sections obtained for each gold layer. In agreement with the results described above, the 25 s gold layer provided the highest number of MS peaks with higher intensity in all the areas of the selected mass range, including the 700–900 Da range (see Fig 2B, 2D and 2F). These results confirm the better performance of the thinner gold nanolayer.

We designed a second Au nanolayer optimization setup considering as starting point the 25 s Au layer, considered optimal in the previous experiment. We applied various sputtered Au layers using deposition times ranging from 15 to 45 s in steps of 5 s onto consecutive sections of liver tissues. The laser power was kept at 60% for all the layers since all of them must be compared in the same conditions. Moreover, we also acquired a tissue section with no Au coating as reference of ionization without Au (0 s). In each case we acquired a complete MSI dataset containing approximately 300 pixels with a pixel size of 100x100 μm.

**Fig 3:**
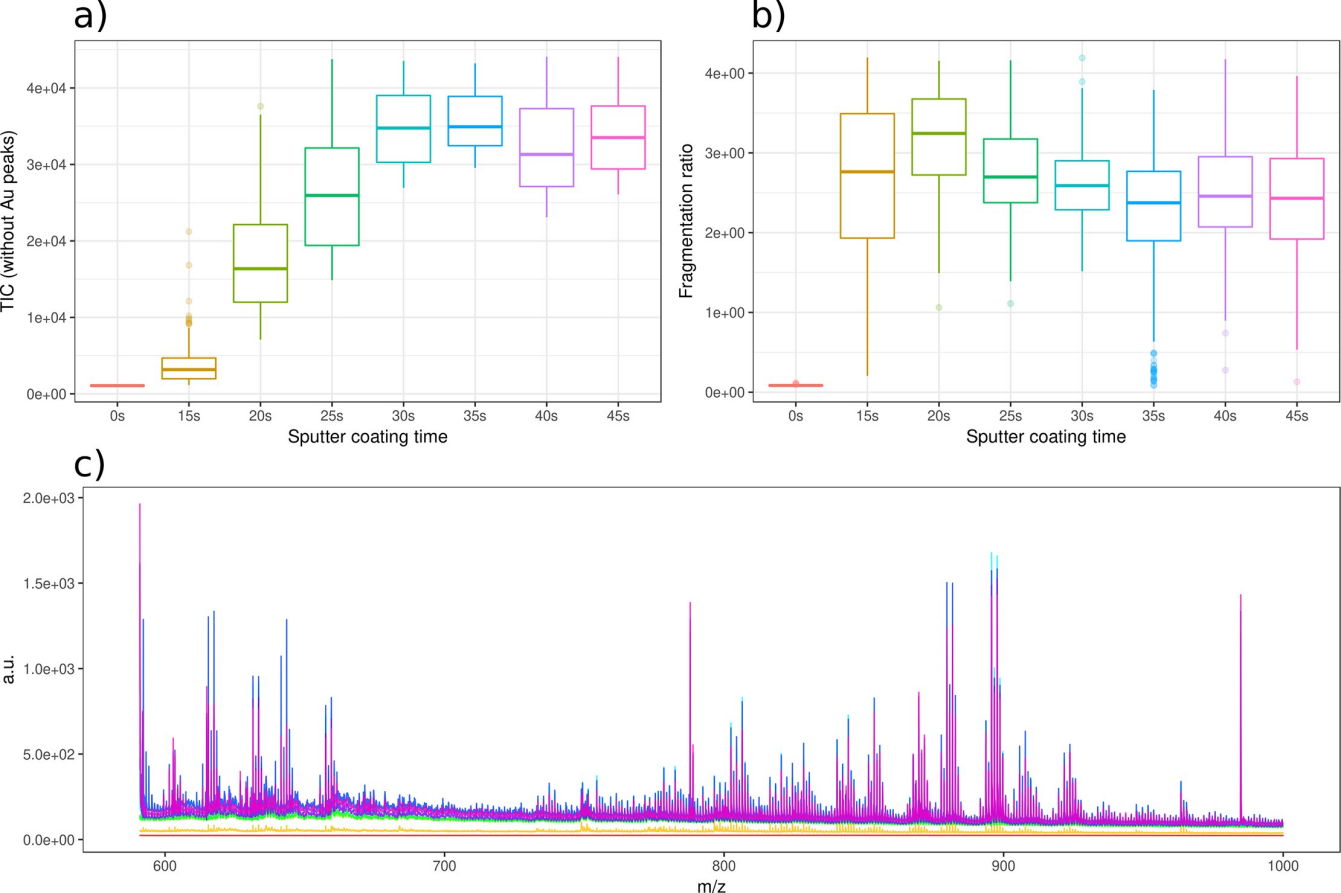
Comparison of various Au coating times MSI performance using 250 pixels of each MS image. **(A)** TIC vs. Au coating time at a laser power of 60%. Au cluster peaks were removed to avoid biasing the experiment since Au MS intensity increases with the sputter coating time. **(B)** Fragmentation ratio of each Au layer was calculated dividing the intensity of to the head group fragment of the phosphatidylcholines (*m/z* 184) peak by the sum of all peak intensities in the 500 to 1000 *m/z*. **(C)** Plot of average spectra from all Au layers with the same coloring as boxplots **(A)** and **(B)**.

After LDI-MS acquisition, the MS data obtained with each of the tested gold layers were compared to determine the optimal gold coating time. As specified in the materials and methods section (section 2.6), spectra were acquired in reflectron positive mode and processed using in-house developed R packages rMSI and rMSIproc. We retained the first 250 most intense pixels of each tissue section for the data analysis. This discards regions of the tissue with holes or bad MS performance and provides the same number of sampling points for each sputtered layer. In order to provide an objective comparison criterion between different sputtering conditions, we have calculated two parameters from each gold layer: the total ion count (TIC) defined as the summing up intensities of all MS peaks; and the fragmentation ratio calculated by dividing the intensity of the head group fragment of the phosphatidylcholines (*m/z* 184.07) by the sum of intensities of the MS peaks found in the 500 to 1000 *m/z* range. The fragmentation ratio is an approximation since several species which not exhibit the *m/z* 184 fragment may exist in the 500 to 100 m/z range. However, the proposed fragmentation ratio is a useful to compare the softness of the ionization process between different Au nanolayers since non-fragmented spices are expected to bias all cases with a similar contribution. For the estimation of these three parameters, we have only considered the peaks of the analyzed liver section with a signal to noise ratio (S/N) over 5, excluding the MS peaks of the gold clusters (*m/z* 196.97, 393.93, 590.90, 787.87 and 984.83). Fig 3 shows the results of comparison of the gold deposition times tested. As can be seen in Fig 3A, the highest TIC was obtained with the 35 s gold layer. Moreover, the lowest fragmentation ratio value was also obtained for the 35 s. Fig 3C confirms that the 35 s coating time provides the optimal Au layer since higher peaks were detected with same laser conditions in the 500 to 1000 *m/z* range. The TIC and fragmentation data dispersion is displayed in the boxes height for Fig 3A and 3B. This dispersion is related to pixel-to-pixel intensity variations due to the inherent tissue morphology.

Besides the comparison of various sputter coating times, we also acquired an MS image of a liver tissue section using a 0 s sputter time (all other steps identical) as negative control. The TIC, fragmentation ratio and the average spectrum of this dataset can be seen in Fig 3. Regarding TIC and average spectrum, no MS signals could be retrieved from the 0 s sputter coated tissue section at the same laser fluence as used for the gold coated tissue sections. As a consequence, the measured fragmentation ratio is almost null since the head group fragment of the phosphatidylcholines (*m/z* 184.07) was not detected.

Acquisitions in negative ionization mode could enhance the MS signal of some metabolites and, therefore, we have also explored the performance of the sputtered gold layers in this ionization mode. As an example, Figure A in S1 File shows the average MS spectrum obtained with a 35 s gold layer of a consecutive section of the liver used for the tests in positive mode, with the same laser conditions. A total of 298 MS peaks, 33 of them in *m/z* 700–900 range, were detected in negative mode with an S/N>5 and TIC 2.63x10^5^. Gold peaks were clearly identified in the negative spectrum enabling the internal calibration process also for this ionization mode. These results demonstrate the suitability of gold sputtered layers to acquire MS images in negative ionization mode, opening a wide range of possibilities for specific applications like the analysis of low-weight acids [36] or fatty acids [37]. Nevertheless, in this study we have focused on positive ionization mode as the deposition was optimized for use in this mode.

The results above demonstrate that the best LDI-MS performance was obtained with the 35 s gold layer. This short gold deposition deposited enough gold particles to ensure the desorption/ionization of the metabolites, but also allowed the laser to easily reach the tissue surface. Moreover, the Au cluster peaks were detected with enough intensity to provide for a reliable mass calibration. Longer gold coating times may prevent the proper desorption/ionization of the underlying metabolites because of the dissipation of more laser energy into the thicker gold layer before reaching the tissue surface.

### Au nanolayer characterization

**Fig 4:**
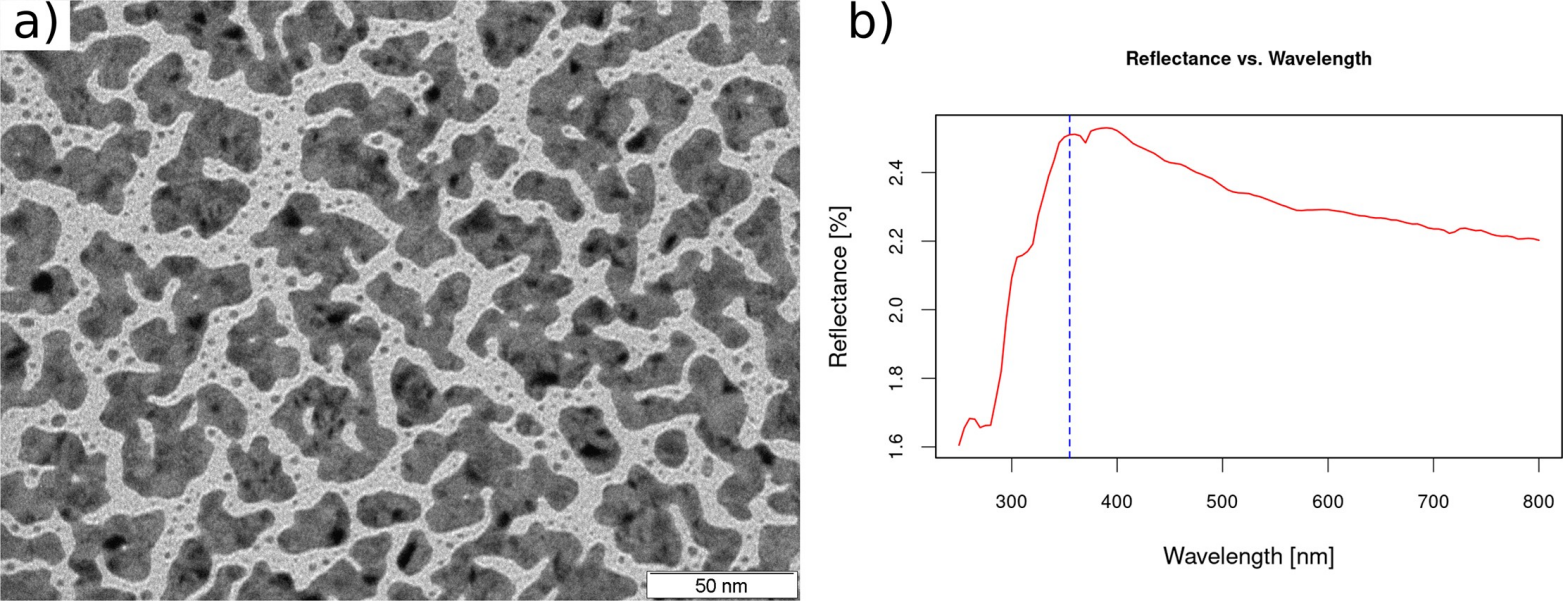
**(A) TEM image at magnification of 400,000 of the optimized gold nanolayer, sputtered in RF mode at 60 W and ambient temperature for 35 s.** The gold nanolayer is represented by the dark grey and black areas. **(B) Reflectance spectrum of the sample system formed by a ITO-coated glass slide, a 10 μm mice brain section and the optimized gold layer.** The vertical dashed blue line corresponds to the Nd:YAG laser wavelength (355 nm) used for the LDI-MS acquisitions.

The morphology of the RF-deposited gold layer was characterized by Transmission Electronic Microscopy (TEM). Fig 4A shows the TEM image of the optimized gold nanolayer deposited over a TEM grid at a magnification of 400,000. TEM images could only be taken by coating a TEM grid and could present a different morphology compared to the gold layer sputtered over a biological tissue. Nevertheless, TEM images could be used as reference. As can be seen in Fig 4A, the gold nanolayer (represented by the dark grey and black areas) is discontinuous. This gold nanolayer forms irregular nanoislands, surrounded by free spaces with a dimension between 5 and 10 nm. A pixel integration over the TEM images showed that the gold particles covered approximately the 65% of the sputtered surface. The sputtered layer over a biological tissue might adapt to the roughness and morphology of the different tissue surfaces, and might be more discontinuous. Although there was a discontinuity of the deposited gold at the nanoscale level, the layer is homogeneous at the LDI-MSI acquisition scale (μm-scale) and therefore, it does not affect the reproducibility of the MSI analysis.

As commented above, one of the most important features of the surfaces developed for LDI-MS applications is the ability to absorb the maximum energy at the wavelength of the instrument laser beam (355 nm for this study). To characterize the performance of the optimized gold nanolayer, we have measured the absorption spectrum of an optimized gold coated, 10 μm tick mouse brain tissue section mounted on a ITO-covered glass slide. This absorption spectrum was measured using a Vis-UV spectrometer with a light incidence angle of 30° in order to mimic as much as possible the acquisition conditions of the laser configuration in the UltrafleXtreme MALDI-TOF instrument [38]. Under the acquisition conditions, the light reflection of the sample system was ca. ~2.5% at 355 nm, which indicates that the tissue-Au-layer system absorbs most of the laser energy achieving high optical efficiency. Fig 4B shows the obtained reflectance spectrum.

## Results: MSI of animal tissues with gold-sputtered layers

The viability of the optimized gold nanolayers for metabolomics MSI applications by LDI-MS was checked by acquiring MSI data of different animal organ tissue sections. The deposition of the optimized gold layer in RF mode, under highly controlled conditions, allowed the acquisition of MSI data using a lower attenuation laser power (60%). C57/BL mouse brain was also used to test the spatial resolution of the method. Fig B in S1 File shows results of LDI-MSI analyses acquired at a spatial resolution of 10 and 20 μm. As an example, one ion was manually selected to show the highly detailed morphological structure in the corpus callosum. In these images we were able to reproducibly reveal small brain tissue structures, demonstrating the capabilities of the sputtered gold layer for high spatial resolution LDI-MSI. In addition, the nanoscale structures observed by TEM demonstrate the high homogeneity of the gold nanolayer from the MSI instrument point of view. Therefore, this enable the MSI acquisitions at very high lateral resolutions being only limited by the laser spot size, although, longer acquisitions times and higher spectrometer sensitivity is needed. Recently, MSI instrument manufacturers started to create new platforms capable of very high lateral resolutions as is the case of Bruker’s RapiFlex. This establishes our gold nanolayer as valuable technique for the future of MSI.

The MS ionization performance of the optimized gold nanolayer was validated on liver and brain tissue sections by comparing the detected MS signals with and without gold nanolayer coating. We set up two consecutive tissue sections from both tissues (liver and brain) mounted side by side on the same ITO-coated glass slide. Then, we covered with a mask the tissue sections used as negative controls. The ITO-coated glass slide was placed in the sputtering chamber and the uncovered tissue sections coated with the optimized gold nanolayer whilst the negative control tissue sections remained in the same experimental conditions except for the gold coating. Then, we performed the MSI acquisition of both tissue sections in the same experimental run and using the same MALDI parameters. Many MS signals revealing liver or brain tissue morphology were obtained within the gold coated tissue, but no MS signals representing morphological structures were detected within the tissue without the gold nanolayer. Here, we could increase the laser power for the uncoated tissue sections in order to retrieve some MS signals. However, we kept the same laser power for all tissue to demonstrate that in the same experimental conditions the ionization efficiency is largely enhanced by our gold nanolayer. To better demonstrate the performance of the optimized gold nanolayer against the no-gold layer, we reconstructed some specific ion images using TIC normalization (see Figures C and D in S1 File). In all cases, a low MS intensity and no tissue morphology were observed in the negative control tissue sections without gold coating. On the other hand, high MS intensity signals and many morphological structures were revealed for the tissue sections analyzed after coating with the optimal gold nanolayer (35 s). The overall MS intensity of MS images with and without gold coating were compared using their average mass spectra. No MS peaks were obtained in the average spectrum of the tissue sections acquired without the gold nanolayer using the same MALDI parameters. This data is available in Figure E in S1 File.

**Fig 5:**
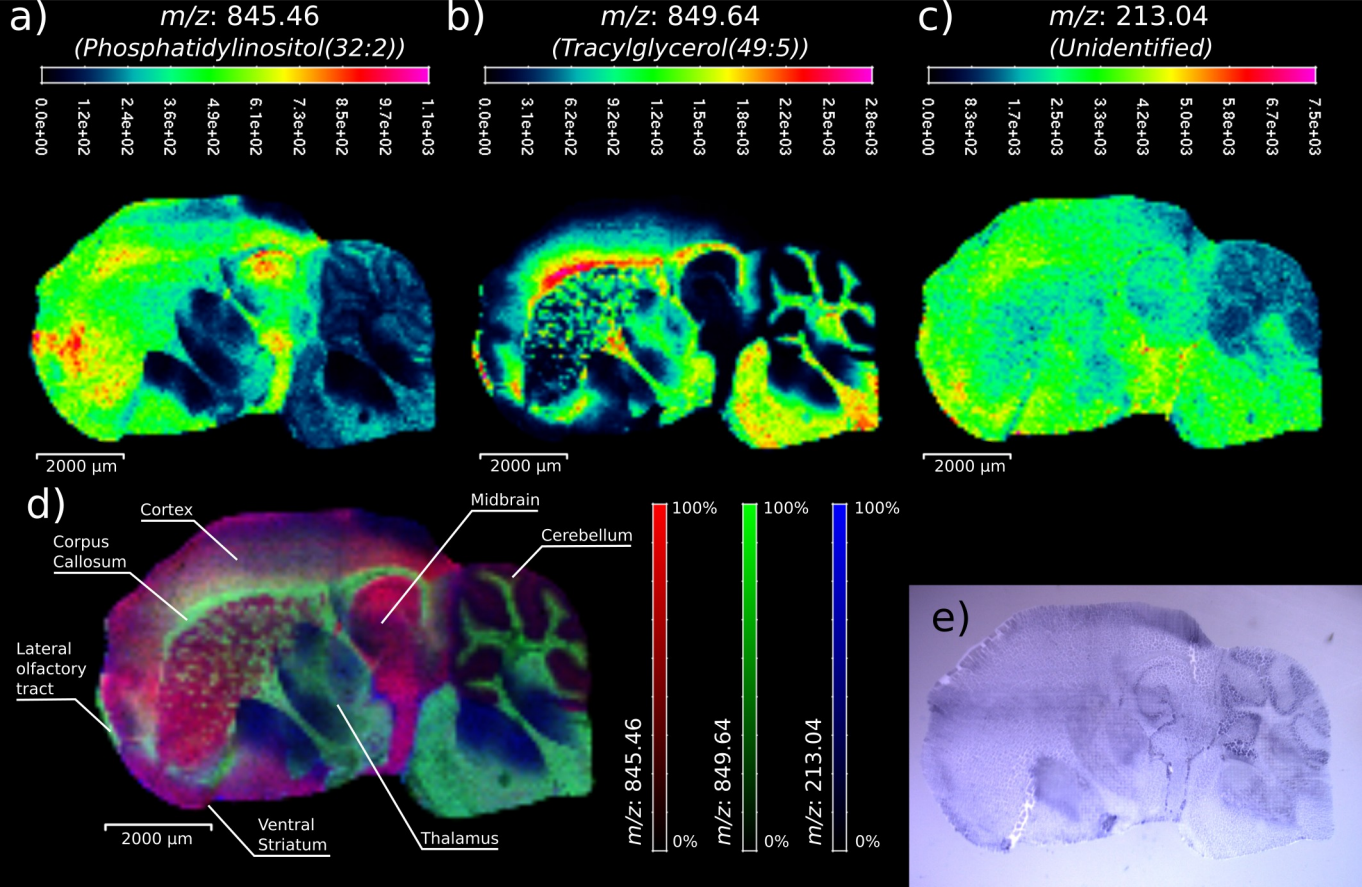
Sagittal section of a mouse brain acquired with the optimized sputtered gold layer at a pixel size of 80 μm. Figures **A**, **B** and **C** plots the relative abundance of three ions found to reproduce the brain morphology (845.46 Da, 849.64 and 213.04, respectively). **D** shows the combined RGB color encoded representation of the three ions that plots different brain areas of the sagittal section. Some of the identified brain regions are labeled. **E** Optical image of a consecutive brain section slice stained with a Hematoxilyn.

In this study, C57/BL mouse brain tissue was acquired with a pixel sizes of 80 μm, a resolution previously reported to be sufficient to reveal the tissue structures in these organs [29]. Moreover, we verified that with this pixel size the laser shots did not overlap and thus detection sensitivity was not compromised. Fig 5 shows the MSI visualizations of three selected ions (*m/z* 845.46, *m/z* 849.64 and *m/z* 213.04, Fig 5A, 5B and 5C, respectively) obtained from a sagittal section of a mouse brain. These figures represent the relative abundance of the selected ions in the color scale showed in each figure, where red represents the areas with maximum ion signals and dark blue the minimum ion signals. As can be seen, the selected ions present different region selectivity in the mouse brain. Fig 5D plots the combined image of these three ions using the RGB color scheme (*m/z* 845.45 in red, *m/z* 849.64 in green and *m/z* 213.04 in blue). In this figure, different brain regions can be clearly distinguished and labeled. The reliability of the developed MSI method was confirmed by comparing the brain morphology obtained with the MSI images with the same brain slice stained with Hematoxilyn (Fig 5E), stained shortly after the MSI acquisition (note the gold layer is porous, which allows the hematoxylin to stain the underlying tissue). In contrast to matrix assisted LDI, the tissue staining can be done without performing any washing step. The ions at *m/z* 845.46 and 849.64 were tentatively identified as the potassium adducts of two lipids commonly found in brain tissues (see Table 1 for further details).

**Table 1:** Putative identification of metabolites in the brain tissue section including the chemical name, ion formula, the experimental *m/z* obtained in our experiment (a), the *m/z* calculated from the database (b), and the mass error of the identification in ppm.

Name	Ion formula	m/z _exp_^a^	m/z _calc_^b^	Δm/z (ppm)
Citrulline	[C_6_H_13_N_3_O_3_+Na]^+^	198.0864	198.0849	-7.8
DAG (35:0)	[C_38_H_74_O_5_+H+NH_4_]^+^	314.7974	314.7971	0.8
Monoacylglycerol (18:2)	[C_21_H_38_O_4_+K]^+^	393.2330	393.2402	18.3
**Cholesterol**	**[C**_**27**_**H**_**46**_**O+Na]**^**+**^	**409.3409**	**409.3441**	**7.8**
**Cholesterol**	**[C**_**27**_**H**_**46**_**O+K]**^**+**^	**425.3091**	**425.3180**	**21.0**
Palmitoyl glucuronide	[C_22_H_42_O_7_+Na]^+^	441.2787	441.2823	8.2
Palmitoyl glucuronide	[C_22_H_42_O_7_+K]^+^	457.2581	457.2562	-4.0
dimethylphosphatidylethanolamine	[C_41_H_78_NO_8_P+Na]^+^	766.5271	766.5357	11.2
**Phosphatidylcholine(34:4)**	**[C**_**42**_**H**_**76**_**NO**_**8**_**P+K]**^**+**^	**792.4857**	**792.4940**	**10.5**
**Phosphatidylserine(34:3)**	**[C**_**40**_**H**_**72**_**NO**_**10**_**P+K]**^**+**^	**796.4646**	**796.4525**	**-15.2**
**Phophatidylethanolamine(40:10)**	**[C**_**45**_**H**_**70**_**NO**_**8**_**P+Na]**^**+**^	**806.4772**	**806.4731**	**-5.0**
Phosphatidylserine(36:5)	[C_42_H_72_NO_10_P+K]^+^	820.4617	820.4525	-11.2
Phosphatidylcholine(38:3)	[C_46_H_88_NO_7_P+Na]^+^	820.6139	820.6191	6.4
**Phosphatidylserine(36:4)**	**[C**_**42**_**H**_**74**_**NO**_**10**_**P+K]**^**+**^	**822.4692**	**822.4682**	**-1.2**
Phosphatidylcholine(38:1)	[C_46_H_90_NO_7_P+Na]^+^	822.6361	822.6347	-1.8
Phosphatidylserine(38:7)	[C_44_H_72_NO_10_P+Na]^+^	828.4711	828.4786	9.1
**Phophatidylethanolamine(42:11)**	**[C**_**47**_**H**_**72**_**NO**_**8**_**P+Na]**^**+**^	**832.4840**	**832.4888**	**5.8**
3,4-dihydroxy-5-all-trans-decaprenylbenzoate	[C_57_H_85_O_4_+H]^+^	834.6539	834.6526	-1.5
Phosphatidylglycerol(38:4)	[C_44_H_79_O_10_P+K]^+^	837.5056	837.5042	-1.7
Phosphatidylcholine(38:2)	[C_46_H_90_NO_7_P+K]^+^	838.6038	838.6086	5.8
Phosphatidylserine(38:7)	[C_44_H_72_NO_10_P+K]^+^	844.4639	844.4525	-13.5
**Phosphatidylinositol(32:2)**	**[C**_**41**_**H**_**75**_**O**_**13**_**P+K]**^**+**^	**845.4620**	**845.4577**	**-5.1**
**Glucosylceramide**	**[C**_**48**_**H**_**91**_**NO**_**8**_**+K]**^**+**^	**848.6360**	**848.6376**	**1.9**
Tracylglycerol(49:5)	[C_52_H_90_O_6_+K]^+^	849.6369	849.6369	0.0
DMPE(40:10)	[C_47_H_74_NO_8_P+K]^+^	850.4673	850.4784	13.0
Phosphatidylcholine(40:2)	[C_48_H_94_NO_7_P+Na]^+^	850.6576	850.6660	9.9
Phosphatidylserine(40:9)	[C_46_H_72_NO_10_P+Na]^+^	852.4730	852.4786	6.5
Phosphatidylcholine(40:3)	[C_48_H_92_NO_7_P+K]^+^	864.6167	864.6243	8.7
Phosphatidylcholine(40:1)	[C_48_H_94_NO_7_P+K]^+^	866.6282	866.6399	13.5
Phosphatidylserine(40:9)	[C_46_H_72_NO_10_P+K]^+^	868.4617	868.4525	-10.6

**Fig 6:**
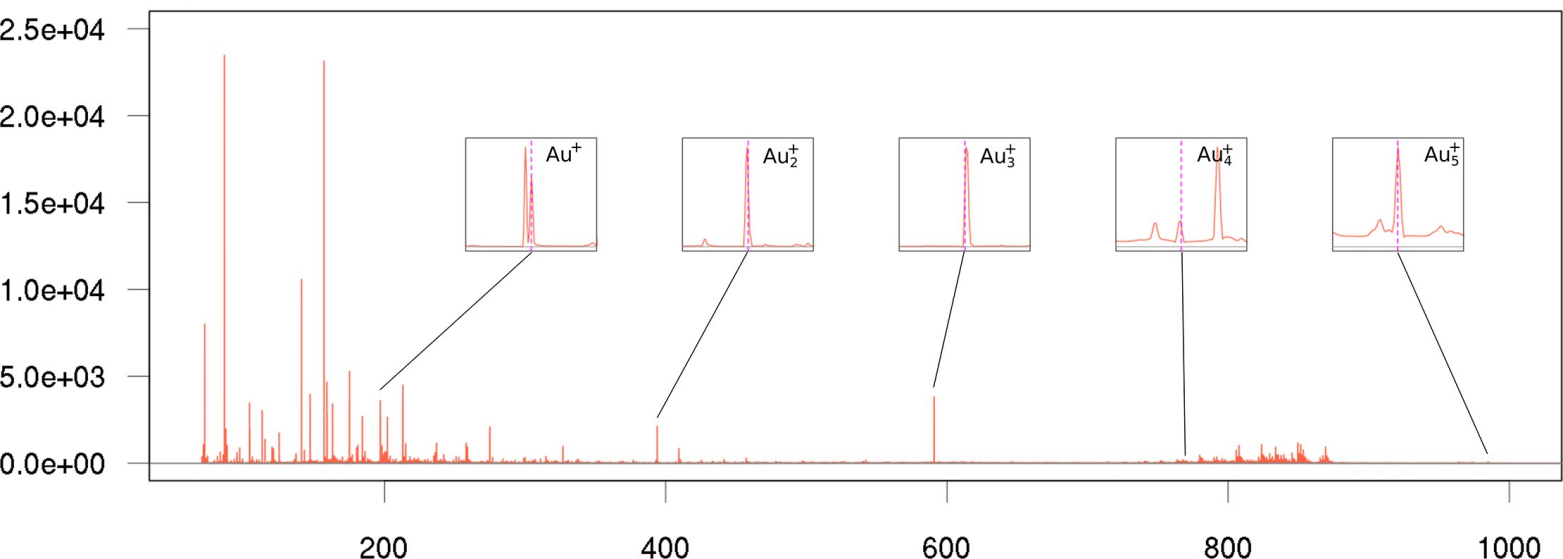
Average MS spectrum of a mice brain section. The MS peaks of gold used for the spectra mass calibration are also indicated.

Fig 6 shows the average MS spectrum from the gold coated mouse brain tissue section. The gold peaks used for the calibration of this spectrum are also indicated. As can be seen, the gold nanolayers are able to promote the desorption/ionization of different metabolites throughout a wide mass range. A total of 356 peaks were detected with a S/N>5, with a TIC of 2.63x10^5^.

The detected peaks were tentatively assigned on the basis of mass accuracy, by matching the experimental mass with the Human Metabolome Data Base (HMDB) [35] database (see section 2.7 for further details). Thirty endogenous metabolites have been tentatively assigned with an identity in the brain section, listed in Table 1, with a mass error below 15 ppm for most metabolites. The list of putative assignments includes amino acids, carbohydrates and other small metabolites and several kinds of lipids, such as fatty acyls, glycerolipids, glycerophospholipids, sphingolipids and sterol lipids. The presented list of tentatively assigned ions is more focused on the *m/z* 700 to *m/z* 900 due to the ion assignment process is more confident in this mass range. The assignation process in the lowest *m/z* range is hampered mainly due to two factors: lower TOF mass accuracy and overlapping fragmented ions. Nevertheless, our method is able to ionize hundreds of compounds in the lowest *m/z* range. This enables the possibility to acquire metabolomics MS images using high mass resolution instruments combined with the developed sputtered Au technique. In bold we have marked the metabolites previously reported in brain by the HMDB to give more confidence to the assignments. To check the accuracy of the identifications we studied the spatial distribution of the ions identified as cholesterol. Cholesterol was detected as sodium and potassium adducts (*m/z* 409.33 and *m/z* 425.31, respectively). As seen in Fig F in S1 File, the spatial distribution of cholesterol ions is similar, thus corroborating that both ions come from the same metabolite. These coherent distributions of cholesterol reinforce the suitability of the gold-induced ionization for MSI.

The optimized gold-induced ionization system presents several advantages regarding other MSI sample preparation techniques. On the one side, sputtering allows the deposition of high purity gold nanoparticles (>99.995%) avoiding contamination of the sample and, therefore, the presence of interfering peaks in the MS spectra. Furthermore, since gold only presents one stable isotope there is no additional dilution of the ion current (as occurs with silver assisted LDI), less mass spectral congestion and the mass spectral peaks can be assigned identities more easily. Furthermore during the MSI data acquisition the mass calibration of the instrument can drift; the presence of gold cluster ions in each pixel facilitates the alignment and calibration between pixels, thus offering a more reliable identification of the metabolites. In this study we have achieved a mass accuracy below 15 ppm for most compounds using gold clusters for internal calibration together with the alignment and calibration software developed in our group[34]. The higher mass errors are associated to instrumental mass resolving power limitations that hampers the separation of compounds with similar molecular weights. Overlapping MS peaks are common in complex samples like biological tissues when analyzed without a previous chromatographic separation stage. This results in a reduction of mass accuracy for TOF instruments preventing to obtain mass errors in the range of 5 ppm as described for non-overlapping MS signals[39].

Compared with the wet deposition of gold layers or organic matrices, the sputtering deposition process used here is known as a fast and highly reproducible technique. The total time needed for the gold layer deposition over a tissue section is around 15 min including sample mounting, pumping the vacuum chamber and deposition. A recent study suggested gold as possible universal material for LDI-MS analysis and imaging [26] because of the high detectability and high mass determination accuracy achieved with this material. The detection of MS peaks in a wide *m/z* range obtained in brain also confirms the potentialities of the application of this new MSI methodology in clinical diagnostics. The rapid and reproducible dry deposition of gold optimized here would promote the use of gold for MSI applications, without the metabolite delocalization inherent to wet deposition methodologies.

## Conclusions

In this study we present the development of an alternative method for the acquisition of MSI data based on the sputter deposition of gold nanolayers over thin tissue slices. Gold is a highly stable material and neither degradation nor oxidation occurs after sample preparation or during the LDI-MS acquisition. The presented sample preparation method is fast, fully automated and reproducible. Furthermore, this dry deposition method avoids the delocalization of metabolites in biological tissues improving the spatial resolution (down to 10 μm, and only limited by the laser configuration) of the obtained MS images.

We were able to obtain optical microscopy images by H&E staining the gold-coated tissue section after the MSI acquisition. This proves that our method is very reliable compared to other tissue-preserving methods since the MSI lateral resolution we obtain outperforms technologies such as DESI-MSI. In future, it could be interesting to explore the combination if our methodology with other MSI techniques using the same tissue section to take advantage of multimodal MSI approaches.

The capability of gold nanoparticles to promote the LDI process has been demonstrated by providing a comparison of the detected MS signals obtained with and without the optimized gold nanolayer. The fact that no MS signal was retrieved without our gold coating protocol reveals the high performance of gold as a suitable material for MSI applications. The capacity of gold to acquire a wide range of metabolites has been demonstrated through the acquirement of MS images of brain tissue. The mass spectra obtained from the analyzed tissues are very rich in the *m/z* range under 1000 Da. Background MS peaks from gold nanoparticles are just five single signals homogenously distributed across the spectra. These signals have a minimal interference on metabolites detection and can also be used for a reliable spectrum alignment and mass calibration between pixels. Moreover, we have been able to tentatively identify thirty endogen metabolites in brain demonstrating the reliability of the acquired spectra. Therefore, the gold-assisted sputtering MSI method presented here could open up new possibilities for a reliable use of MSI in clinical diagnostics. The presented technique will surely find many MSI applications due to the high potential it has demonstrated. Future work should be carried out to establish this technique for MSI. In this regard, the gold layer thickness should be optimized in each specific MSI applications. Moreover, efforts should be made to completely understand the ionization process involved with Au nanoparticles.

## Supporting information

S1 File (Figure A) Average spectrum of a mouse liver section acquired in reflectron negative mode using the 35 s sputter coated gold layer. (Figure B) Mouse brain tissue section acquired high spatial resolution using a Bruker MALDI-TOF/TOF rapifleX instrument. (Figure C) TIC normalized MS images of two manually selected ions at m/z 577.51 and 895.68 of two consecutive mouse liver tissue sections. (Figure D) TIC normalized MS images of two manually selected ions at m/z 866.58 and 868.40 of two consecutive mouse brain tissue coronal section. (Figure E) Average spectra of complete MS images for liver tissue dataset. (Figure F) MS images of ions m/z 409.33 and 425.31 of mouse brain tissue section.
(PDF)

## Abbreviations

DC: Direct Current
H&E: Hematoxylin and Eosin
ITO: Indium Tin Oxide
LDI: Laser Desorption/Ionization
MALDI: Matrix Assisted Laser Desorption/Ionization
MS: Mass Spectrometry
MSI: Mass Spectrometry Imaging
NP: Nano Particle
RF: Radio Frequency
SALDI: Surface Assisted LDI
S/N: Signal to Noise Ratio
TEM: Transmission Electronic Microscopy
TIC: Total Ion Count
TOF: Time Of Flight
UV: Ultra Violet
