# Supplementary material for: Assessing the potential of sputtered gold nanolayers in mass spectrometry imaging for metabolomics applications
Source: PLoS One. 2018 Dec 12;13(12):e0208908. doi: 10.1371/journal.pone.0208908 (PMC6291137; doi:10.1371/journal.pone.0208908)
Supplement: S1 File — (Figure A) Average spectrum of a mouse liver section acquired in reflectron negative mode using the 35 s sputter coated gold layer. (Figure B) Mouse brain tissue section acquired high spatial resolution using a Bruker MALDI-TOF/TOF rapifleX instrument. (Figure C) TIC normalized MS images of two manually selected ions at m/z 577.51 and 895.68 of two consecutive mouse liver tissue sections. (Figure D) TIC normalized MS images of two manually selected ions at m/z 866.58 and 868.40 of two consecutive mouse brain tissue coronal section. (Figure E) Average spectra of complete MS images for liver tissue dataset. (Figure F) MS images of ions m/z 409.33 and 425.31 of mouse brain tissue section. (PDF) [file pone.0208908.s001.pdf]

Assessing the potential of sputtered gold nanolayers in mass spectrometry imaging for metabolomics applications

Supporting information

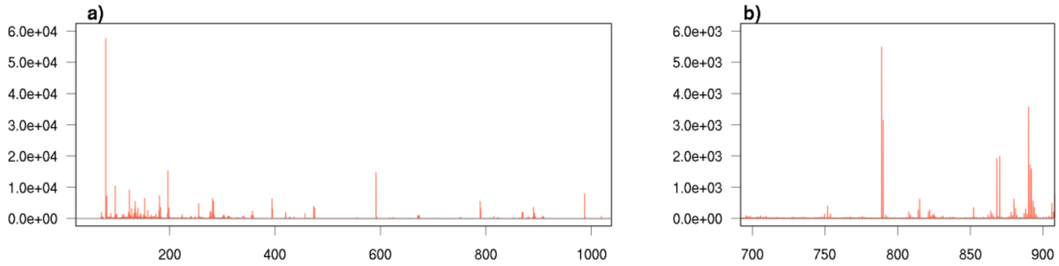

Fig A. Average spectrum of a mouse liver section acquired in reflectron negative mode using the 35 s sputter coated gold layer. **a)** The full MS spectrum until *m/z* 1000 and **b)** Zoom of the MS spectrum between *m/z* 700 and 900.

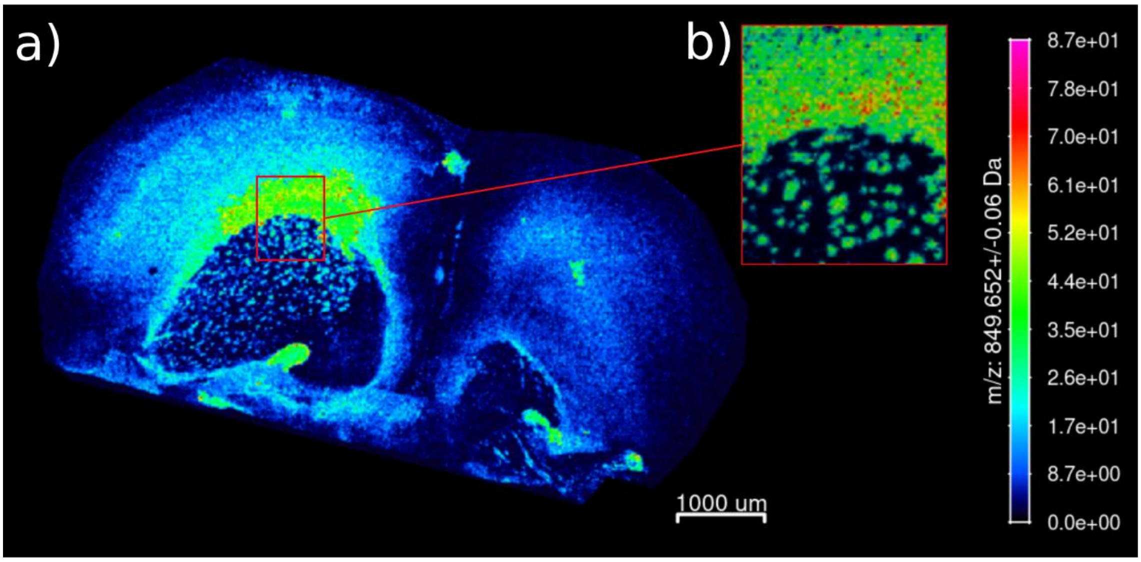

Fig B. Mouse brain tissue section acquired high spatial resolution using a Bruker MALDI-TOF/TOF rapifleX instrument. **a)** Complete brain coronal section acquired at 20 µm. **b)** A small part of corpus callosum and striatum acquired at 10 µm using the same tissue section.

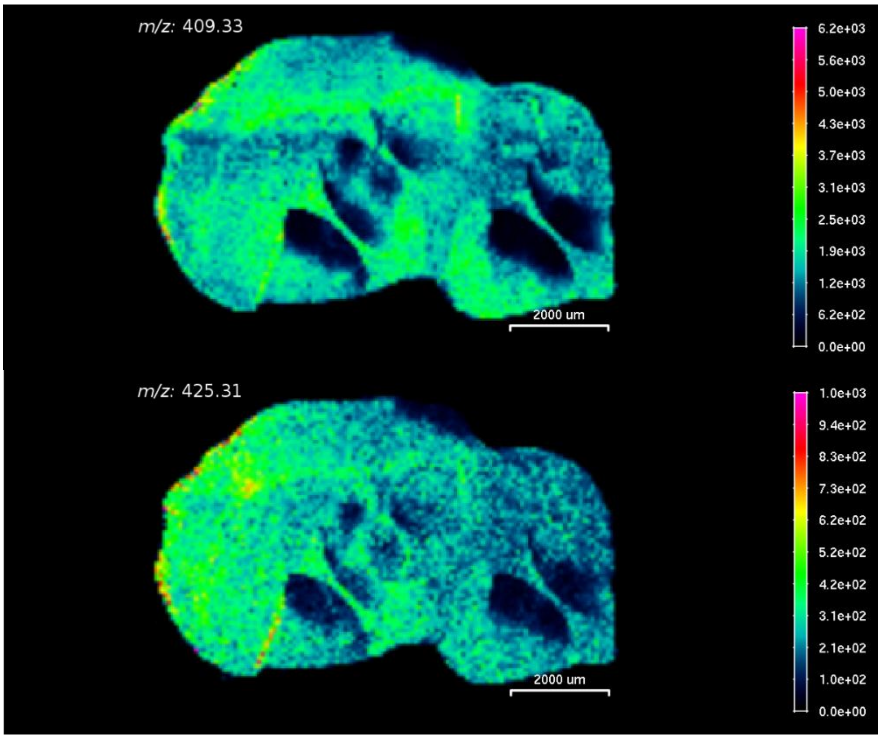

Fig C. MS images of ions *m/z* 409.33 and 425.31 of mouse brain tissue section. These ions presents two highly correlated images that have been putatively assigned as cholesterol ($[C_{27}H_{46}O+Na]^+$ and $[C_{27}H_{46}O+K]^+$ respectively).

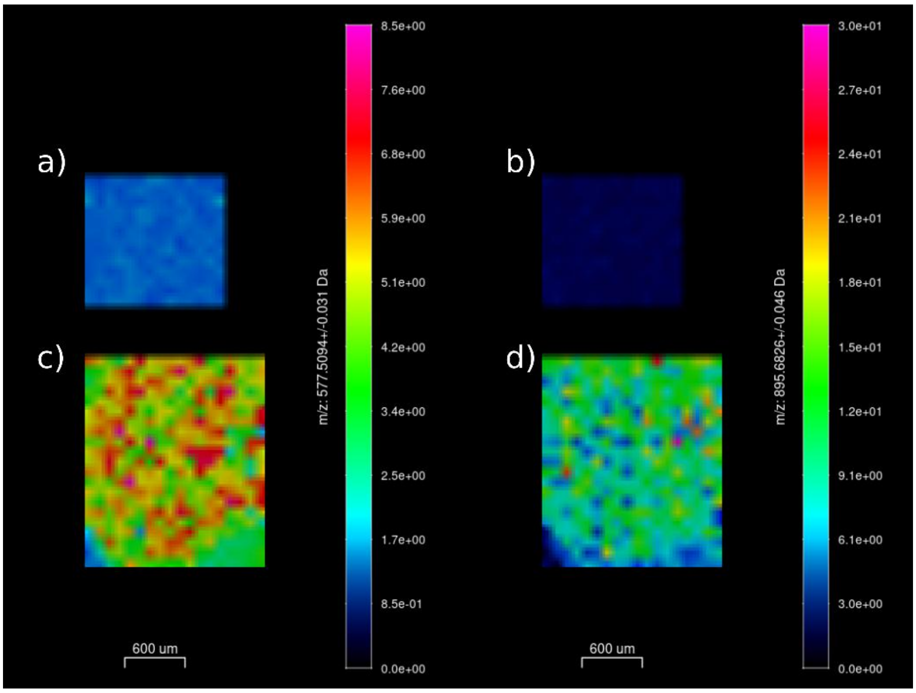

Fig D. TIC normalized MS images of two manually selected ions at *m/z* 577.51 (**a, c**) and 895.68 (**b**, **d**) of two consecutive mouse liver tissue sections. Images **a**) and **b)** corresponds to a tissue section acquired without the Au nanolayer. Images **c)** and **d)** represent the same ions obtained with the optimized sputtered gold nanolayer. Both MSI datasets were acquired at the same experimental run and with the same MALDI instrument parameters.

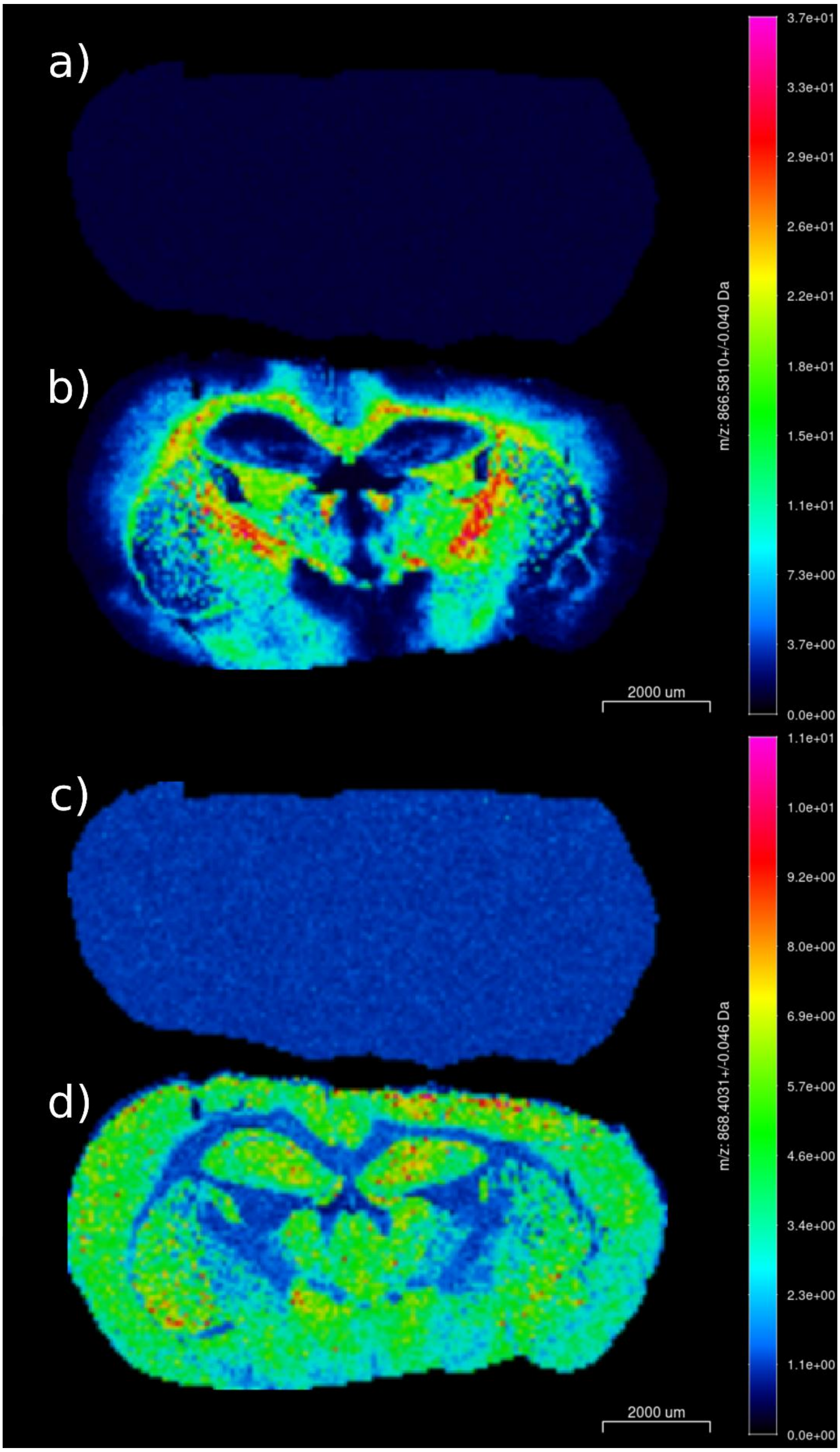

Fig E. TIC normalized MS images of two manually selected ions at *m/z* 866.58 **a), b)** and 868.40 (**c**, **d**) of two consecutive mouse brain tissue coronal section. Images **a**) and **c)** corresponds to a tissue section acquired without the Au nanolayer. Images **b)** and **d)** represent the same ions obtained with the optimized sputtered gold nanolayer. Both MSI datasets were acquired at the same experimental run and with the same MALDI instrument parameters.

a)

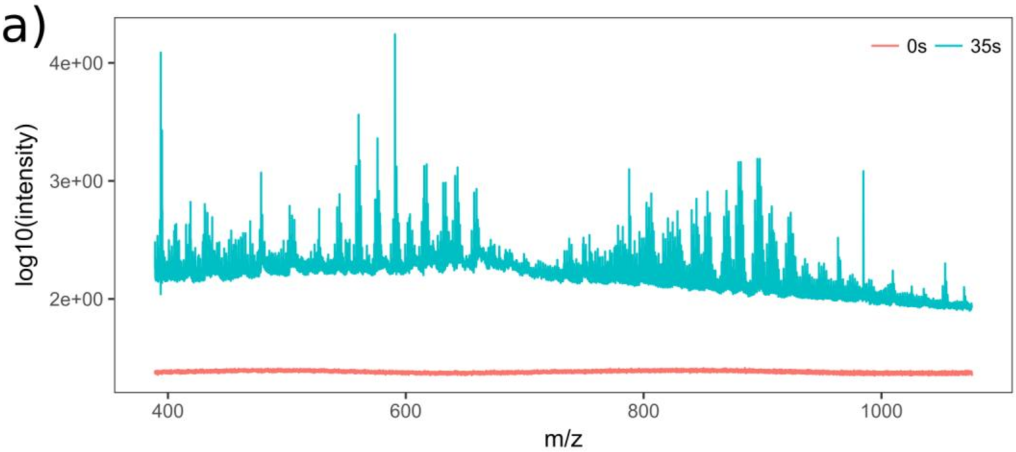

b)

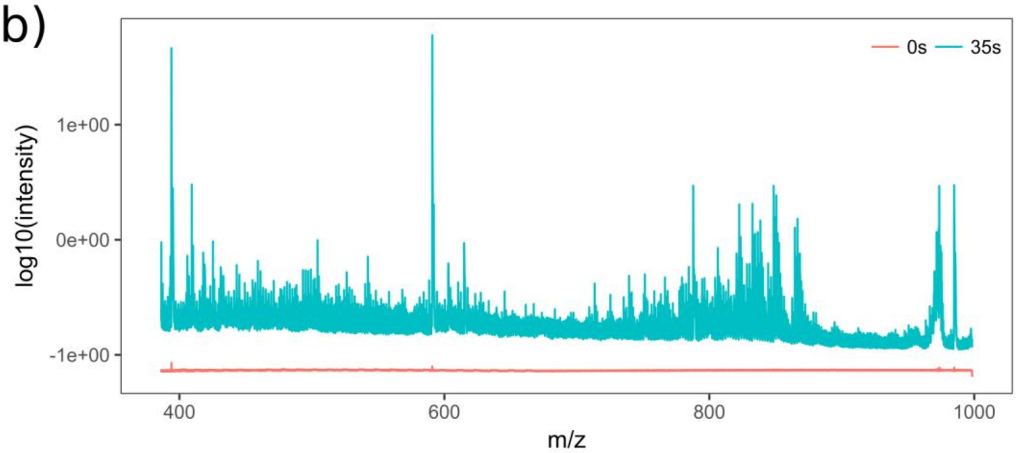

Fig F. Average spectra of complete MS images for liver tissue dataset (ion images are in Fig. S4). a) and the coronal brain sections dataset (ion images are in Fig. S5) **b)**. The average spectrum of an MS image without gold coating (0s) is compared against the average spectrum obtained at the next section coated with the optimized Au nanolayer (35s). The intensities of average spectra have been logarithmically scaled since the overall MS intensity of the tissues sections without the gold layer is so low. Both tissue sections (0s vs. 35s) were acquired using exactly the same MALDI settings and in the same experimental run. As it can be seen, no MS signal is obtained without the application of the gold nanolayer. In contraposition, many MS signals are detected from the same tissues with the optimized gold nanolayer.
